# Validity of Self-reported *Helicobacter pylori* Eradication Treatment From Questionnaire and Interview Surveys of the JPHC-NEXT Study: Comparison With Prescription History From Insurance Claims Data

**DOI:** 10.2188/jea.JE20230168

**Published:** 2024-09-05

**Authors:** Tomomi Kihara, Kazumasa Yamagishi, Takuya Imatoh, Hikaru Ihira, Atsushi Goto, Hiroyasu Iso, Norie Sawada, Shoichiro Tsugane, Manami Inoue

**Affiliations:** 1 Department of Public Health Medicine, Institute of Medicine, and Health Service Research and Development Center, University of Tsukuba, Tsukuba, Japan; 2 Ibaraki Western Medical Center, Chikusei, Japan; 3 Division of Cohort Research, Institute for Cancer Control, National Cancer Center, Tokyo, Japan; 4 Department of Health Data Science, Graduate School of Data Science, Yokohama City University, Yokohama, Japan; 5 Bureau of International Health Cooperation, National Center for Global Health and Medicine, Tokyo, Japan; 6 National Institute of Health and Nutrition, National Institutes of Biomedical Innovation, Health and Nutrition, Tokyo, Japan; 7 Division of Prevention, Institute for Cancer Control, National Cancer Center, Tokyo, Japan

**Keywords:** validation, epidemiology, *Helicobacter pylori*, Japanese, claims database

## Abstract

**Background:**

We aimed to evaluate the validity of self-administered questionnaire surveys and face-to-face interview surveys for the detection of *Helicobacter pylori* eradication therapy.

**Methods:**

Participants were a cohort, aged 40–74 years, living in three different locations of Japan, who took part in the baseline survey (2011–2012) of the Japan Public Health Center-based Prospective Study for the Next Generation (JPHC-NEXT). Five years after the baseline survey, a questionnaire and interview survey were independently conducted to determine the history of *Helicobacter pylori* eradication treatment over the 5-year period. Prescription of *Helicobacter pylori* eradication medications in national insurance claims data from the baseline survey to the 5-year survey was used as a reference standard.

**Results:**

In total, 15,760 questionnaire surveys and 8,006 interview surveys were included in the analysis. There were 3,471 respondents to the questionnaire and 2,398 respondents to the interview who reported having received *Helicobacter pylori* eradication treatment within the past 5 years. Comparison of the questionnaire survey to national insurance claims data showed a sensitivity of 95.1% (2,213/2,328), specificity of 90.6% (12,174/13,432), positive predictive value of 63.8% (2,213/3,471), negative predictive value of 99.1% (12,174/12,289), and Cohen’s Kappa value of 0.71. Respective values of the interview survey were 94.4% (1,694/1,795), 88.7% (5,507/6,211), 70.6% (1,694/2,398), 98.2% (5,507/5,608), and 0.74.

**Conclusion:**

Both the questionnaire and the interview showed high sensitivity, high specificity, and good agreement with the insurance claim prescriptions data. Some participants may have received eradication treatment without going through the public insurance claim database, resulting in a low positive predictive value.

## INTRODUCTION

Infection with *Helicobacter pylori (H. pylori)* has been established as the most important cause of gastric cancer,^1^ and *H. pylori* eradication has been recommended by the International Agency for Research on Cancer working group as a key strategy for preventing gastric cancer in high-risk countries.^2^ A recent systematic review targeting Japanese^3^ pointed out that, although the protective effect of *H. pylori* eradication has been observed in healthy or asymptomatic populations, there were many unsolved issues, including adverse effects and appropriate timing of eradication, follow-up intervals after eradication, and cost effectiveness. Further cohort studies with valid information on *H. pylori* eradication status are needed. To estimate the status of *H. pylori* eradication at the population level, it is practical to use self-administered questionnaires and face-to-face interviews, and it is essential to ensure the validity of using these data for exposure assessment in each study population. Through the Japan Public Health Center-based Prospective Study for the Next Generation (JPHC-NEXT), we obtained the self-reported history of *H. pylori* eradication in two ways: a self-administered questionnaire survey (mainly mailed or distributed to home) and a face-to-face interview survey held on-site at health checkups. This study aimed to assess the validity of detecting history of *H. pylori* eradication via self-administered questionnaires and face-to face interviews, methods used in the baseline and follow-up surveys in the JPHC-NEXT, using the prescription history from national insurance claims data as a reference standard. This study is positioned as a validation study, which precedes future investigation regarding unsolved issues on *H. pylori* eradication.

## METHODS

### Study settings

The JPHC-NEXT study is an ongoing community-based cohort study.^4^ The baseline survey was conducted between 2011 and 2016, and 115,385 people from seven areas agreed to participate in the study, including the use of insurance claims database, and completed the lifestyle questionnaire. Some individuals who participated in the health checkups or study-specific sample donation site also provided blood samples. They had their *H. pylori* antibody titer and pepsinogen measured and were informed of the results (47.9% of the participants). Five (±1) years after the baseline survey, a questionnaire survey was administered to the participants that included an item asking about history of *H. pylori* eradication. Some individuals who participated in the health checkups were asked about *H. pylori* eradication during face-to-face interviews. All participants provided written informed consent. This study was approved by the Institutional Review Boards of the National Cancer Center (approval number: 2017-250), Osaka University (2012-072), and the University of Tsukuba (87-11).

### Study population of the validation study

**Figure 1.**
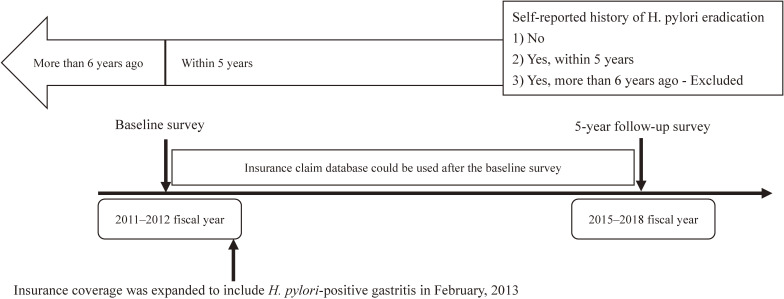
Diagram of the 5-year follow-up survey of self-reported history of *H. pylori* eradication and the available insurance claims data for detection of *H. pylori* eradication treatment Insurance coverage was expanded to include *H. pylori*-positive gastritis in February, 2013

Before February 2013, *H. pylori* eradication therapy was covered under national insurance only for patients with gastric ulcer, duodenal ulcer, gastric mucosa-associated lymphoid tissue lymphoma, idiopathic thrombocytopenic purpura, and post-endoscopic resection of early gastric cancer.^5^^,^^6^ From February 2013, the indications for *H. pylori* treatment were expanded to enable asymptomatic patients to receive eradication treatment through national insurance coverage.^7^ Therefore, this analysis included 41,251 people aged 40–74 years who participated in the baseline survey in 2011 or 2012 fiscal year, before the expansion of insurance coverage, from the three locations in Japan (Chikusei, Saku, and Yokote). Thereafter, the status of eradication from individuals from the questionnaire at the 5-year follow-up survey and/or face-to-face interview in 2015–2018 were collected. We excluded those who were unable to use insurance claims for more than 5 consecutive years, those who did not provide appropriate information on *H. pylori* eradication, and those who reported having received *H. pylori* eradication therapy more than 6 years ago. The temporal context of the 5-year follow-up survey of self-reported history of *H. pylori* eradication and the available insurance claims data for detection of *H. pylori* eradication is shown in Figure 1.

### Questionnaire survey

The 5-year follow-up questionnaire survey was a self-administered instrument. The query about *H. pylori* eradication was “Have you ever received eradication therapy for *Helicobacter pylori*? If yes, please circle the approximate time of the therapy.” The response options were “No”, “Yes, less than 1 year ago”, “Yes, between 1–5 years ago”, and “Yes, more than 6 years ago”.

### Interview survey

Approximately 50% of participants in the JPHC-NEXT came to the health checkup sites or study-specific sample donation sites. They were asked about their *H. pylori* eradication status during face-to-face interviews. The interview began with the question, “Have you ever been tested for *Helicobacter pylori*?”. The next question was “Have you ever received eradication therapy for *Helicobacter pylori*? If yes, please select the approximate time period.” Responses were recorded as “None”, “Less than 1 year”, “Within 1–5 years”, “More than 6 years ago”, or “Don’t know/other’.

### Insurance claims

Insurance claims data were available for participants who were enrolled in the Municipal National Health Insurance, or the Health Insurance for the Elderly, for those aged 75 or over. These two insurance systems cover approximately 40% of the Japanese population.^8^ Employee Health Insurance covers approximately 60% of Japanese (and their family members) employed at large companies^9^; however, these claim data were not available for this study. During the follow-up period, the type of insurance system applicable to the participant may change due to changes in employment status, moving out of the study area or other reasons; therefore, we determined the availability of insurance claim data for each participant based on list of medical insurance subscribers by fiscal year.

We defined a participant with history of *H. pylori* eradication as those who received a prescription for a 1-week supply of any combinations of three medications for eradication during the period from the baseline survey to the 5-year follow-up survey. The combinations of three medication were either single drug combinations or packaged preparations, and included primary eradication therapy (amoxicillin, clarithromycin, and either a proton-pump inhibitor or potassium-competitive acid blocker) and secondary eradication therapy (metronidazole instead of clarithromycin and other drugs are same as primary treatment), which were covered by public medical insurance. Other combinations are not covered by Japanese public medical insurance systems. Drugs are coded with a 9-digit code, starting with 6, for the computer receipt processing system (eTable 1). If the treatment is performed using a combination different from that allowed by public insurance such as for penicillin allergy or if the disease is not covered by insurance, the treatment is performed at the patient’s own expense and, thus, cannot be detected in the insurance claim database.

### Statistical analysis

Sensitivity, specificity, positive predictive value (PPV), negative predictive value (NPV), and Cohen’s kappa statistics with 95% confidence intervals were calculated for the questionnaire and interview surveys using insurance claims data as a reference standard. A kappa value of ≤0.20 was considered as poor, 0.21–0.40 as fair, between 0.41–0.60 as moderate, 0.61–0.80 as good, and 0.81–1.00 as very good.^9^ All analyses were performed using SAS Version 9.4 (SAS Institute Inc., Cary, NC, USA).

## RESULTS

**Figure 2.**
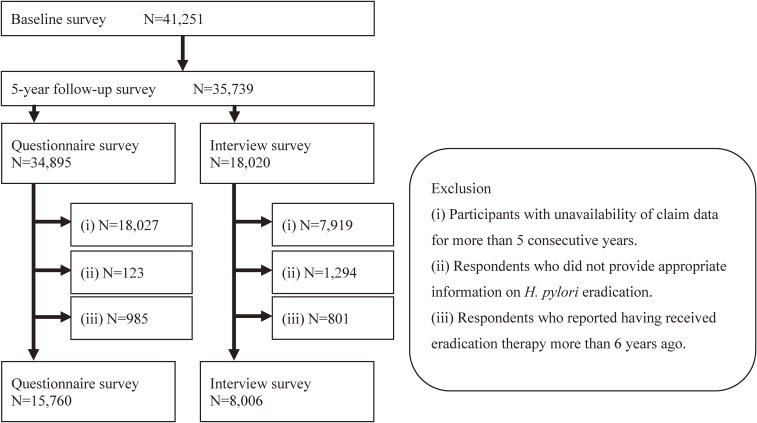
Flow chart for inclusion and exclusion of the participants

Of 41,251 people who participated in the baseline survey, 34,895 responded to the 5-year follow-up survey questionnaire and 18,020 responded to the face-to-face interview at the health checkup site. The final sample size was 15,760 for the questionnaire survey and 8,006 for the interview survey (Figure 2). Among the participants, 8,409 responded to the questionnaire survey only, 655 responded to the face-to-face interview only, and 7,351 responded to both. The mean age of the respondents was 62.9 (standard deviation [SD], 8.2) years for the questionnaire survey and 62.5 (SD, 7.6) years for the face-to-face survey. The percentage of men was 45.0% for the questionnaire survey and 43.3% for the face-to-face interview survey.

A total of 2,579 participants received prescriptions for *H. pylori* eradication therapy at least once between baseline and the 5-year post-survey. Of these, 85.4% received only primary eradication, 2.0% received only secondary eradication, and 12.6% received both.

Setting insurance claims as a reference standard for detecting *H. pylori* eradication therapy administration, questionnaire survey had a sensitivity of 95.1% (2,213/2,328), specificity of 90.6% (12,174/13,432), PPV of 63.8% (2,213/3,471), and NPV of 99.1% (12,174/12,289). Respective values of the interview survey were 94.4% (1,694/1,795), 88.7% (5,507/6,211), 70.6% (1,694/2,398), and 98.2% (5,507/5,608), respectively (Table 1). The questionnaire survey and the insurance claims data had good agreement (κ = 0.71). The face-to-face survey and the insurance claims data also had good agreement (κ = 0.74).

**Table 1.** Overall results for each questionnaire and interview survey using insurance claims data

		Questionnaire survey			Interview survey		
	
		*Helicobacter pylori* eradication within 5 years			*Helicobacter pylori* eradication within 5 years		
	
		+	−	Total	+	−	Total
Eradication prescription in the insurance claims	+	2,213	115	2,328	1,694	101	1,795
	−	1,258	12,174	13,432	704	5,507	6,211
	Total	3,471	12,289	15,760	2,398	5,608	8,006
Age, years, mean (SD)		62.9 (8.2)			62.5 (7.6)		
Male, %		45.0			43.3		
Sensitivity, %		95.1 (94.2–95.9)			94.4 (93.3–95.4)		
Specificity, %		90.6 (90.1–91.1)			88.7 (87.9–89.5)		
Positive predictive value, %		63.8 (62.2–65.4)			70.6 (68.8–72.5)		
Negative predictive value, %		99.1 (98.9–99.2)			98.2 (97.9–98.6)		
Cohen's Kappa Value		0.71 (0.70–0.73)			0.74 (0.73–0.76)		

Data in parentheses are 95% confidence intervals, unless otherwise noted.

The results did not differ substantially across the study areas, although the PPV of questionnaire in Chikusei was slightly lower compared with the other areas (eTable 2). The PPV and Kappa coefficients were higher for those who participated in the baseline survey in 2012 than those in 2011 (eTable 3), probably because insurance coverage was expanded on February 21, 2013.

## DISCUSSION

Both the questionnaire and the interview had good agreement with the insurance claims data, with Kappa coefficients above 0.61. To the best of our knowledge, this is the first study to examine the validity of self-reported *H. pylori* eradication history using a large community-based study covering multiple areas around Japan.

The PPV of self-reports, either by questionnaire or interview, were approximately 60–70%. The reason for the low values could be that: (1) the self-reports for eradication were incorrect to some extent; (2) the prescription was not captured in the insurance claims data because the eradication was performed without insurance coverage, or (3) participants who were received eradication therapy more than 6 years ago may have mistakenly responded that they were received eradication therapy between 1–5 years ago due to misremembering. In our study, the claims data were not available before the baseline survey. A previous study reported that difference between the age of eradication in the medical records and the self-reported age of eradication was within 1 year for about 80% of the participants, but only about a quarter of the participants were in perfect agreement.^10^

The values on validities of the interviews were generally better than those of the questionnaires. Because the interview survey was carried out with health checkup participants, it is possible that more health-conscious people were included in the study. In addition, more accurate answers may have been obtained via the face-to-face interview survey because the interviewers were able to explain the meaning of the questions if the participants had difficulty understanding. Of note, in our questionnaires, we did not annotate the meaning of eradication therapy, and did not ask about history of *H. pylori* tests, which may have caused some misunderstanding among the participants. However, both the questionnaire and the interview showed high sensitivity and specificity, so we assumed that both were useful to ascertain the history of *H. pylori* eradication.

Self-reported medication for hypertension, diabetes, and dyslipidemia, which usually require long-term prescriptions, showed high validity by comparison with insurance claims database (sensitivity 0.84–0.95; specificity 0.97–0.99).^11^ The high sensitivity in the present study suggested that people could easily recall prescriptions even if they were not administered regularly. On the other hand, the lower specificity may be partly because some eradication treatments were performed without insurance coverage and therefore do not appear in the insurance claims database.

Our study has several strengths. First, it has high external validity because of the inclusion of the general population, rather than being focused purely on patients, and the inclusion of multiple regions with the large number of participants. Second, we simultaneously examined the validity of the questionnaire survey and the interview survey on the history *H. pylori* eradication. Some limitations need to be mentioned. First, as mentioned earlier, eradication therapy without insurance coverage is not captured in the insurance claims data. However, insurance coverage was expanded to include *H. pylori*-positive gastritis in February, 2013; therefore, the proportion of persons who received eradication therapy without insurance coverage may be small in this study. Second, the possibility of recall bias should be noted. As a part of the baseline survey, 47.9% of JPHC-NEXT study participants had their *H. Pylori* antibody titer and pepsinogen measured, and were informed of the results, which may have led infected participants to receive eradication therapy. These participants may have easily recalled *H. pylori* eradication when answering the 5-year follow-up survey. Of the 15,760 participants who responded to the 5-year questionnaire and were included in the analysis, 10,248 had been measured for *H. pylori* antibody titer and pepsinogen at baseline. Their PPV (65.5%) and Cohen’s Kappa value (0.72) were slightly higher than those of the other 5,512 participants (57.0% and 0.67, respectively). Third, the claim data of Employee Health Insurance were not available for this study. Although there are no overt differences in the access to medical care by type of insurance system in Japan, the demographic characteristics, such as sex, age, and occupation, differ between those with the Municipal National Health Insurance and those with Employee Health Insurance. Thus, the generalizability of our finding to persons to Employee Health Insurance is uncertain.

### Conclusion

In conclusion, the findings of our study suggest that self-reports, whether using self-administered questionnaires or face-to-face interviews, are reliable to ascertain *H. pylori* eradication treatment, although the interview survey showed slightly better validities than the questionnaire survey. Capturing the history of *H. pylori* eradication treatment in a self-reports survey will allow large-scale, community-based follow-up studies to investigate the impact of *H. pylori* eradication on the development of gastric cancer.

## SUPPLEMENTARY MATERIAL

eTable 1. Codes for the receipt computer processing system in Japan

eTable 2. Area specific results by area for each questionnaire and interview survey using insurance claims as a reference standard

eTable 3. Baseline-year specific results by baseline year for each questionnaire and interview survey using insurance claims as a reference standard
