# Supplementary material for: Validity of Self-reported Helicobacter pylori Eradication Treatment From Questionnaire and Interview Surveys of the JPHC-NEXT Study: Comparison With Prescription History From Insurance Claims Data
Source: J Epidemiol. 2024 Sep 5;34(9):453–7. doi: 10.2188/jea.JE20230168 (PMC11330703; doi:10.2188/jea.JE20230168)
Supplement: Supplementary file 1 [file je-34-453-s001.pdf]

eTable 1. Codes for the receipt computer processing system in Japan

Rabeprazole Sodium (Proton-pump inhibitor)	610412202	622402601	621997201	621999501	621999901
	622001201	622002201	622005301	622007301	622011201
	622012901	622016201	622020602	622021401	622023301
	622025601	622026001	622031601	622032801	622035501
	622040101	622060201	622089501	622118601	622505501
Omeprazole (Proton-pump inhibitor)	610443068	610443069	610443070	610443071	620001980
	620001981	620001983	620001984	620001985	620001986
	620002694	620002695	620003914	621622303	621622403
	621630104	621630502	621630601	621630701	621680901
	621681001	621681401	621794301	621977902	621983103
	622077801	620002696	620005367		
Lansoprazole (Proton-pump inhibitor)	610462010	610462011	612320549	612320550	620002743
	620002744	620002749	620002750	620002871	620002872
	620004087	620004088	620005581	620005583	620005584
	620005585	620007127	620007128	620009451	620009452
	621673701	621673801	621693101	621693201	621743701
	621743801	621780301	621780401	621796301	621796401
	621919001	621919101	622015801	622015901	622023101
	622023201	622034601	622034701	622469801	622469901
Esomeprazole Magnesium Hydrate (Proton-pump inhibitor)	622080701	622080801			
Vonoprazan Fumarate (Potassium-competitive acid blocker)	622404401	622404501			
Amoxicillin Hydrate (Amoxicillin)	610454003	616130039	616130040	616130132	616130295
	620006829	620006919	620006920	620007024	620007025
	620007026	620008584	620009117	621073001	621073901
	622054901	622066501	622111101	622112902	622127801

	622165902				
Clarithromycin	616140102	616140105	620003926	620003927	620003928
	620003929	620003930	620003931	620003932	620003933
	620003934	620003935	620003946	620004076	620006670
	620008013	621742103	621752901	622079401	
Metronidazole	620007057				
Lansoprazole, Amoxicillin Hydrate, Clarithromycin (LANSAP)	610462048	610462049			
Lansoprazole, Amoxicillin Hydrate, Metronidazole (LAMPION Pack)	622029101				
Rabeprazole Sodium, Amoxicillin Hydrate, Clarithromycin (Rabecure PACK)	622289101	622289201			
Rabeprazole Sodium, Amoxicillin Hydrate, Metronidazole (Rabefine PACK)	622289301				
Vonoprazan Fumarate, Amoxicillin Hydrate, Clarithromycin (VONOSAP Pack)	622485401	622485501			
Vonoprazan Fumarate, Amoxicillin Hydrate, Metronidazole (VONOPION Pack)	622485601				

eTable 2. Area specific results by area for each questionnaire and interview survey using insurance claims as a reference standard

	Questionnaire survey			Interview survey		
Area	Chikusei	Saku	Yokote	Chikusei	Saku	Yokote
Number of participants	1,677	11,106	2,977	1,313	4,037	2,656
Age, years, mean (SD)	60.4 (7.5)	63.2 (8.5)	63.1 (7.2)	60.2 (7.5)	62.9 (7.9)	63.1 (7.0)
Male, %	43.4	46.1	42.0	40.8	44.6	42.5
Sensitivity, %	96.6 (94.6–98.7)	94.6 (93.5–95.8)	95.4 (93.6–97.2)	95.3 (92.6–98.0)	94.3 (92.9–95.7)	94.0 (91.9–96.2)
Specificity, %	86.0 (84.2–87.8)	91.6 (91.1–92.2)	89.3 (88.1–90.5)	90.4 (88.6–92.1)	86.7 (85.4–87.9)	90.5 (89.3–91.8)
Positive predictive value, %	59.9 (55.5–64.3)	64.1 (62.1–66.0)	65.3 (62.0–68.7)	68.2 (63.2–73.2)	72.4 (70.0–74.7)	68.1 (64.5–71.7)
Negative predictive value, %	99.2 (98.7–99.7)	99.1 (98.9–99.3)	98.9 (98.5–99.4)	98.9 (98.2–99.5)	97.6 (97.1–98.2)	98.6 (98.1–99.1)
Cohen’s Kappa Value	0.67 (0.63–0.71)	0.72 (0.70–0.74)	0.72 (0.69–0.75)	0.74 (0.70–0.79)	0.74 (0.72–0.76)	0.74 (0.70–0.77)

SD, standard deviation.

Data in parentheses are 95% confidence intervals, unless otherwise noted.

eTable 3. Baseline-year specific results by baseline year for each questionnaire and interview survey using insurance claims as a reference standard

	Questionnaire survey		Interview survey	
Baseline year	2011	2012	2011	2012
Number of participants	6,606	9,154	3,437	4,569
Age, years, mean (SD)	61.7 (8.6)	63.7 (7.8)	61.3 (7.9)	63.5 (7.3)
Male, %	45.1	45.0	43.5	43.1
Sensitivity, %	95.1 (93.7–96.5)	95.0 (93.9–96.1)	93.1 (91.3–94.9)	95.3 (94.0–96.5)
Specificity, %	90.4 (89.6–91.2)	90.8 (90.2–91.5)	87.1 (85.8–88.4)	89.9 (88.9–90.9)
Positive predictive value, %	60.4 (57.8–63.0)	66.0 (64.0–68.0)	66.5 (63.6–69.4)	73.8 (71.5–76.2)
Negative predictive value, %	99.2 (98.9–99.4)	99.0 (98.8–99.2)	97.9 (97.3–98.5)	98.4 (98.0–98.9)
Cohen's Kappa Value	0.69 (0.66–0.71)	0.73 (0.71–0.75)	0.70 (0.67–0.73)	0.77 (0.75–0.79)

SD, standard deviation.

Data in parentheses are 95% confidence intervals, unless otherwise noted.
